# Fabrication of Blood Capillary Models for Live Imaging Microarray Analysis

**DOI:** 10.3390/mi11080727

**Published:** 2020-07-27

**Authors:** Muhammad Asri Abdul Sisak, Fiona Louis, Sun Hyeok Lee, Young-Tae Chang, Michiya Matsusaki

**Affiliations:** 1 Department of Applied Chemistry, Graduate School of Engineering, Osaka University, 2-1 Yamadaoka, Suita, Osaka 565-0871, Japan; a-sisak@chem.eng.osaka-u.ac.jp; 2 Joint Research Laboratory (TOPPAN) for Advanced Cell Regulatory Chemistry, Graduate School of Engineering, Osaka University, 2-1 Yamadaoka, Suita, Osaka 565-0871, Japan; f-louis@chem.eng.osaka-u.ac.jp; 3 Center for Self-assembly and Complexity, Institute for Basic Science (IBS), Pohang 37673, Korea; tnsgur02@postech.ac.kr (S.H.L.); ytchang@postech.ac.kr (Y.-T.C.); 4 School of Interdisciplinary Bioscience and Bioengineering, Pohang University of Science and Technology (POSTECH), Pohang 37673, Korea; 5 Department of Chemistry, Pohang University of Science and Technology (POSTECH), Pohang 37673, Korea

**Keywords:** blood capillary models, fibrin gels, collagen microfibers

## Abstract

Conventional microarray analysis usually deals with the monolayer or two-dimensional (2D) assays for the high-throughput screening applications. Even though these cell-based assays are effective for preliminary screening at least to have information on cytotoxicity, they do not adequately re-create the in vivo complexity of three-dimensional (3D) tissues. In this study, 3D-blood capillary models were constructed by using physiological collagen microfibers (CMF), which provide the extracellular matrix in the complex tissue. Micro-droplets of fibrin gels containing CMF, endothelial cells, and fibroblasts were cultured for five days in 48-wells plate to provide a medium-throughput system for screening applications. Blood capillaries networks were formed by optimizing the concentration of CMF used and the number of cells. Finally, this screening method was a powerful assay for the application on the selection of not only a specific chemical probe for blood capillary live-imaging, but also a drug, aptamer, and peptide with potential blood vessel targeting property.

## 1. Introduction

The regular method of screening compounds in the field of drug discovery begins with the two-dimensional (2D) cell culture-based assays, followed by animal model assays, before reaching the clinical trials [1]. However, in these methods, many of the drugs fail during clinical trials, which might be due to a lack of clinical efficacy and/or an unacceptable toxicity [2]. Even though the 2D cell-based assays are effective for preliminary screening at least to have information on cytotoxicity, they sometimes provide misleading and non-predictive data for in vivo responses due to their unnatural microenvironment [3]. This means that cells could not maintain their original organ functions and morphologies in conventional 2D cell culture systems. Therefore, the creation of a technique for constructing a three-dimensional (3D) tissue having a structure and function similar to a living body in vitro is strongly demanded and it could also provide more reliable data for various applications.

To fulfill the demand in the medical field, research on constructing a tissue having such 3D structure in vitro has been actively conducted in order to form a model as similar as possible to the living body [4]. Numerous studies on fabrication techniques of blood capillary models have been reported, such as bio-printing, organ-on-chip or microfluidic device, electrospinning techniques, cells sheet, as well as scaffold structure [5,6,7,8,9,10,11]. However, some problems remain, such as the inability to control of the geometry of the three-dimensional arrangement or the biocompatibility: even in 3D bioprinted scaffolds it is still difficult to precisely tune their endothelialization after adding the cells and some synthetic vascular grafts for clinical application were found to be associated with thrombosis, aneurysm, and intimal hyperplasia occurrence due to their lack of compliance (possibility to strain/expanse or contract) [12]. The electrospinning method has another limitation, which is the low cell invasion of the scaffolds, generally due to a too small pore size, and the cell sheet is challenged by the difficulty to keep the tissue integrity and its low mechanical strength of the stacked cell sheet tissue [6]. All of these limit their use as a tissue model for high-throughput assay. For instance, organ-on-chip technique which is strictly designed for in vitro applications is not suitable for the high-throughput system due to its complicated handling requirements and their biological issues due to the sources of the cells that are limited and of high cost (e.g., primary cells or induced pluripotent stem cell, iPSc) [13]. Hydrogels also show the ability to fabricate the 3D-tissue models, owing to their biocompatibility and mechanical properties [6,14,15]. Recently, photo-crosslinkable hydrogels in particular were developed, collagen-poly (ethylene glycol) diacrylate (PEGDA) hydrogel, for instance, providing cell-adhesive and tunable mechanical strength characteristics, suitable for vascular engineering applications [14]. Therefore, there is still a need for an in vitro, human tissue model that is simple, accessible, reproducible, and quick to produce which could fulfill the demand for the microarray analysis.

Generally, tissues are composed of cells and extracellular matrix (ECM). The ECM is a complex 3D matrix that guides cell adhesion, proliferation, differentiation, morphology, and gene expression [16]. While several synthetic components were already used for recreating engineered vasculature, natural components are generally found to be more suitable, especially in the form of hydrogel, being similar in nature to ECM. Fibrin is one of the examples of natural biomaterial used in tissue engineering which derives from the native ECM [17,18]. Fibrin is also one of the natural polymers that has been used in vascular tissue models for various applications such as implantation [19] and tissue engineering [20,21,22]. The main advantages of using natural materials to support cells in tissue models are that these molecules are naturally recognized by cells and they are more probable to stimulate physiological responses in cells such as cells migration, proliferation, and remodeling of the matrix.

In our previous study, we have fabricated a spheroid called tissue balls using collagen microfibers (Figure S1a). However, it has a limited observation ability due to the thickness (>500 µm) and the high turbidity of the tissue balls. Nonetheless, the fact to use collagen microfibers dispersion instead of classic collagen gels made by dilution allows to reach a final high concentration which is more similar to in vivo. This fibers form also allows to provide a physiologic environment for the cells, allowing forces and energy transmissions, as well as biological signals regulating cells functional responses, especially for vasculature formation through integrin-induced adhesion of endothelial cells [23]. Therefore, the solution for these issues lead to a thinner and more transparent tissue in order to overcome the thickness and turbidity issues, respectively. Here, we fabricated the 3D-blood capillary models using collagen microfibers (CMF) [23,24,25,26], where fibrin acts as a tissue scaffold. A micro-droplet of tissue that consists of a coculture of HUVEC and fibroblast in an optimized amount of CMF with a constant concentration of fibrinogen and thrombin were seeded on each of the wells to be cultured in 48-well plate and serve as a microarray analysis for the screening of chemical probes as an example of application.

## 2. Materials and Methods

### 2.1. Preparation and Characterization of the Collagen Microfibers (CMFs)

For the preparation of collagen microfibers (CMFs), we used the method described in our previous study [26]. Briefly, collagen I sponge (kindly donated by Nippon Ham Foods Ltd. Osaka, Japan) was crosslinked in a vacuum dryer (HD-15H, iLW, Osaka, Japan) and then incubated at 200 °C for 24 h. Subsequently, crosslinked collagen I (10 mg/mL in Milli-Q water) was homogenized (Violamo VH-10 homogenizer, AS ONE, Osaka, Japan. S10N-10G with 10 mm diameter and 115 mm length probe) for 6 min and subsequently sonicated (VC50, Sonics and Materials, Newtown, CT, USA) for 20 s/cycle (100 cycles) with cooling in an ice bath for 10 s, resulting in smaller size fragments of collagen. The dispersion was filtered by a 42 μm nylon mesh (PA-42μ, AS ONE, Osaka, Japan) and then freeze-dried (Freeze dryer FDU-2200, Eyela Co., Tokyo, Japan) for three days. The obtained CMF was kept in a desiccator at room temperature until use. The average length of CMF was measured from the phase contrast microscopy image while using ImageJ software (version 1.53a, National Institute of Health, Bethesda, MD, USA). The CMF was named as CMF-200 and CMF-50, as the average length was obtained at around 197 ± 35.6 µm and 47 ± 12.8 µm for the CMF before and after the sonication step, respectively.

### 2.2. Fabrication of the 3D-Blood Capillary Models in 48-Well Plate

For the preparation of 3D-blood capillary models, we used the method described in our previous study [23]. Briefly, normal human dermal fibroblast (NHDF, LONZA, Basel, Switzerland) and human umbilical vein endothelial cells (HUVECs, LONZA, Basel, Switzerland) were trypsinized (5 min, 37 °C) and then centrifuged at 1000 rpm for 5 min at room temperature for collecting the cells by removing the supernatant. 5 U/mL of thrombin (T4648, Sigma-Aldrich, St. Louis, MO, USA) was mixed with cells (NHDF and HUVEC) in Dulbecco modified Eagles’s medium (DMEM, 08458, High-Glucose, Nacalai tesque, Kyoto, Japan) and regarded as Solution 1. Subsequently, Solution 2 comprising 15 mg/mL of CMF-50 mixed with 5 mg/mL of fibrinogen (F8630, Sigma-Aldrich, St. Louis, MO, USA) in DMEM (FBS free). Thus, the 10 μL of fibrin gels containing 0.15 mg of CMF-50, 1.5 × 10^4^ of NHDF and 7.5 × 10^3^ of HUVECs (or green fluorescent protein labeled HUVEC/GFP-HUVEC for studying vascular network formation in live imaging analysis) were seeded in each well of 48-well plate. After that, the tissue drops were incubated at 37 °C for 30 min to allow the gelation process upon the reaction of fibrinogen and thrombin. The steps were repeated for different concentrations of CMF (0, 5, 10, 20, and 25 mg/mL) and different volumes of seeded drops [5 µL (2.5 µL solution 1 + 2.5 µL solution 2) and 30 µL (15 µL solution 1 + 15 µL solution 2)]. The number of cells for different volumes of seeded drops was also changed [5 µL (7.5 × 10^3^ of NHDF and 3.75 × 10^3^ of HUVECs) and 30 µL (4.5 × 10^4^ of NHDF and 2.25 × 10^4^ of HUVECs). Finally, the tissues were cultured for five days at 37 °C, 5% CO_2_, with mixed cell culture medium, which is DMEM and Endothelial Cell Growth Medium-2 (EGM-2, LONZA, Basel, Switzerland) at ratio 1:1. The mix culture medium was changed every two days.

### 2.3. Immunofluorescence Staining and Histological Analysis

After five days of culture, the tissues were fixed with 4% paraformaldehyde (PFA, 30525-89-4, Wako Pure Chemical Industries, Osaka, Japan) for 20 min at room temperature (RT). Then, the tissues were evaluated in order to know the capillary network formation using immunofluorescence staining of the endothelial cell surface marker: CD31. First, the cells in the tissue were rinsed 3× in phosphate-buffered saline (PBS, D5652, Sigma-Aldrich, St. Louis, MO, USA) and permeabilized with 0.2% Triton-X 100 (234729, Sigma-Aldrich, St. Louis, MO, USA) in PBS for 20 min at RT. Next, 1% of bovine serum albumin (BSA, A3294, Sigma-Aldrich, St. Louis, MO, USA) in PBS was added into the tissue for 60 min in order to block the unspecific staining of the antibody. After washing 3× with PBS, the tissues were incubated with the CD31 primary antibodies (1:100, Dako, Hamburg, Germany, M0823, diluted in 1% BSA in PBS) overnight at 4 °C. Later, the tissues were washed 3× with PBS and then the secondary antibody, Alexa 647 (anti-mouse, 1:200 in PBS with 1% BSA), mixed together with Hoechst 33342 (H3570, Thermo Fisher Scientific, Whaltam, MA, USA) for nuclei staining (1:1000 in PBS with 1% BSA), was added and incubated for 1 h at RT. The images were obtained while using a confocal laser scanning microscopy (CLSM, CQ1 from Yokogawa Corporation, Tokyo, Japan). For histology analysis, the fixed 3D tissues were washed 3× with PBS and then sent to the Applied Medical Research company (Osaka, Japan) for paraffin embedding and immunohistochemistry. Sectioned 3D tissues were stained with hematoxylin eosin (HE) or CD31 staining. The images were obtained using a FL EVOS Auto microscope (Thermo Fisher Scientific, Whaltam, MA, USA) for measuring the tissue thickness and observing the lumen of the capillaries.

### 2.4. Analysis of Blood Capillaries Formation

The mean fluorescence intensity of the total area (2D image) of the blood capillaries was measured by ImageJ software in order to quantify blood capillary formation. The fluorescence intensity was given by the fluorescence image of the CD31-antibody (specific marker for endothelial cells) bounded with its secondary antibody (Alexa 647, Thermo Fisher Scientific, Whaltam, MA, USA). In addition, total branching points (indicates the connecting point of the blood capillaries) were analyzed by Imaris software (Version 9.2.1,Bitplane, Belfast, UK), while using the “Filament Algorithm” function.

### 2.5. Application of Microarray Analysis for Screening of Chemical Probes

Eighty of near-infrared (NIR) chemical probes with the core structure of cyanine with different substituents (R group) were screened to select the highest specific probe binding to the blood capillaries [27]. The dried probes were dissolved in DMSO in order to have 10 mM of stock solution. For the experiment, the probes were further diluted in DMEM to get a final concentration of 1 µM, including 0.1% of final DMSO concentration, known to be not cytotoxic for the cells. After 20 min of probe incubation at 37 °C, 5% CO_2_, on the blood capillary models, the tissues were then washed with PBS to remove the unbound probes. Live imaging of probes adsorption was next acquired by confocal laser scanning microscopy (CLSM, CQ1). The images were finally used to analyze the colocalization of the probes to the blood capillaries.

### 2.6. Statistical Analysis

All of the data are presented as mean ± standard deviation (s.d.) from three independent experiments, unless otherwise specified. Statistical comparisons between groups were analyzed while using two-tailed Student’s *t*-tests. A *p*-value * < 0.01 and ** < 0.001 was considered to be statistically significant. *N.S.* indicates no significant difference. Statistical analysis were also performed using EzAnova software (version 0.98, University of South Carolina, Columbia, SC, USA) with Tukey multiple comparison test (one-way ANOVA). A *p*-value * < 0.05, ** < 0.01 and *** < 0.001 was considered to be statistically significant.

## 3. Results and Discussion

### 3.1. D-Blood Capillary Models in 48-Well Plate

In the previous study, the 3D-blood capillary models were fabricated by coculture between NHDF and HUVEC and seeding together with CMF in 96-well non-adherent plate (modified method from [24,25]). Subsequently, the mixtures were centrifuged to undergo the sedimentation process of the cells and the CMF. After one day of culture in the 96-well, it resulted in a sphere shape (called as tissue ball), as shown in Figure S1a. However, as we observed the blood capillary networks of the tissue balls (2 mm of ball diameter) by CLSM, the images of blood capillaries (as illustrated in Figure S1a) were not possible to fully observe, especially in the center of the tissue, which might be due to the limitation of the CLSM, unable to scan a thickness of more than 500 µm. Besides that, the high turbidity of the tissue balls also limits the penetration of laser through the tissue. Only tissue clarifying can increase the lens working distance, but it is not compatible with live imaging [28]. Thus, the solution was to produce a thinner and more transparent tissue.

In this study, a 3D blood capillary model was fabricated using fibrin gel, which could be the key to provide a thinner and less turbidity tissue for live imaging in order to address the issues from the previous tissue balls models. The CMF was also further sonicated after the homogenization process to get smaller fragments (CMF-50), which allow for overcoming the issue of aggregation occurring with the bigger size CMF-200 (Figure S1b), resulting in the less formation of blood capillaries [27]. Therefore, CMF-50 was further used for the fabrication of 3D-blood capillary models.

**Figure 1:**
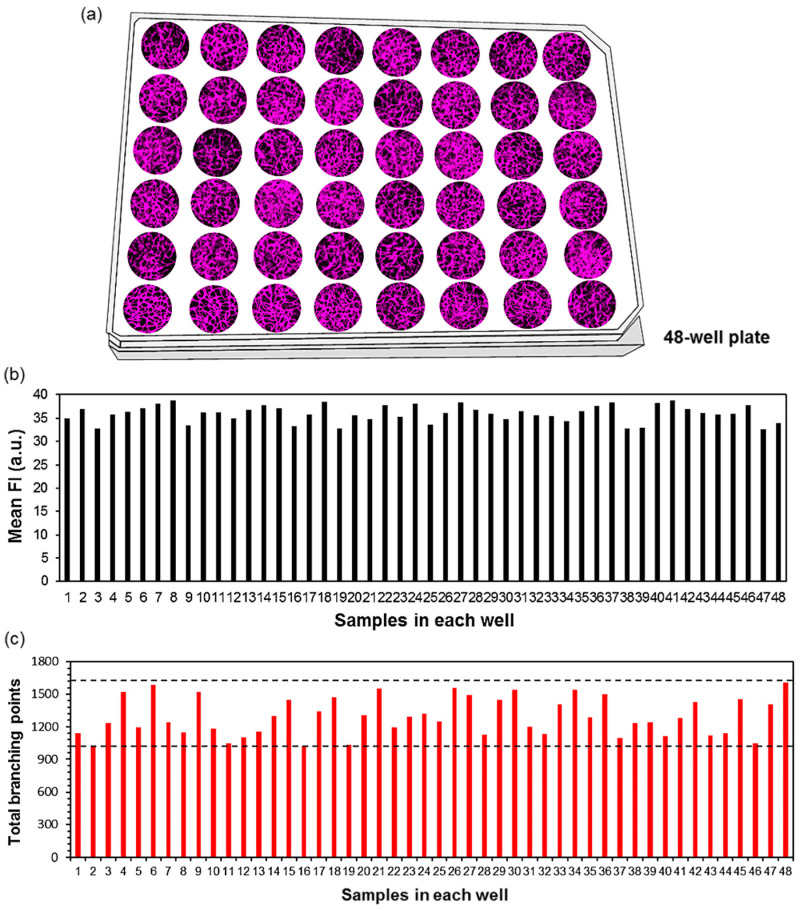
Assessment of the reproducibility of the three-dimensional (3D) model of blood capillary to be used as a microarray assay. (**a**) Representative CD31 immunostaining (endothelial marker) of the 48 different 3D-blood capillary models constructed in the medium-throughput 48-well plate illustration. (**b**) Mean fluorescence intensity of each sample in 48-well plates. (**c**) Total branching points from each of the 3D-blood capillary models (48 samples). Dotted lines indicate the range of the branching points of all samples in the plate (1020–1620) to validate the quality of each sample.

The next step was to optimize the seeding process. As mentioned in the methods part, two solutions containing different compounds need to be mixed to activate the gelation process. The different possible sequences of order for the mixing of the two solutions were thus compared by dropping first the Solution 1 (cells and thrombin) and then Solution 2 (CMF and fibrinogen) and the opposite way. As shown in Figure S2a, the tissues with the order of Solution 1 on Solution 2 gave a homogenously capillary network as compared to the heterogeneous network on the tissues with the order of Solution 2 on Solution 1 (Figure S2b). In addition, the importance of the mixing step after dropping the two solutions for the tissues was checked, resulting in an empty capillary network area in the middle of the tissues, probably due to a heterogeneous aggregation of the CMF in the center of the drop tissues. Hence, we chose the sequence with the order of Solution 1 to Solution 2, without mixing, for the further experiments. The validity of this method for microarray analysis was characterized by its culture inside a 48-well plate to serve as medium-high throughput microarray analysis. The quantification of each sample for the mean fluorescence intensity of their CD31 endothelial marker and its total branching points using ImageJ and Imaris software, respectively. The 3D-blood capillary models were successfully cultured and observed by CLSM (Figure 1a) in a reproducible way. For the mean fluorescence intensity (Figure 1b), the results showed that all 48 samples of a 48-wells plate had a similar fluorescence intensity (36.0 ± 1.8). Concerning their total branching points (Figure 1c), which indicate the complexity of the network formation, was successful found in the range of 1020 to 1620 (1292.2 ± 174.9), as measured by Imaris software.

### 3.2. The Effect of Different Amounts of CMF in the 3D-Tissues

**Figure 2:**
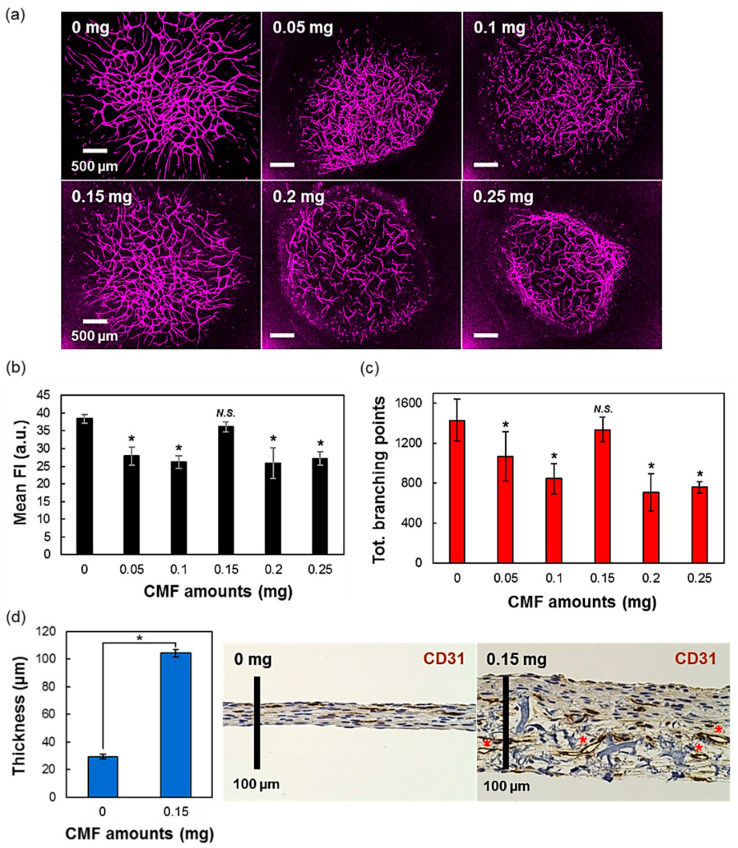
The effects of using different amounts of collagen microfibers (CMFs) in fibrin gels. (**a**) Confocal images of 3D-blood capillary models with different amounts of CMF (0, 0.05, 0.10, 0.15, 0.20, and 0.25 mg). The blood capillaries were immunostained by CD31 antibody and fluorescence using Alexa Fluor 647. (**b**) Quantification data for mean fluorescence intensities and their (**c**) total branching points for each sample in different CMF amounts. (**d**) Histology data showing the open lumen (red asterisk) and the graph of thickness between 3D tissue of blood capillary using 0 mg and 0.15 mg of CMF, respectively. Data are presented as mean ± s.d. (*) indicates that data are significantly different (*p* < 0.01). *N.S.* indicates no significant difference.

Before obtaining the reproducibility of the Figure 1 model, we also investigated the effect of different amounts of CMF in the 3D-blood capillary models and optimized it. Different amounts of CMF (0, 0.05, 0.1, 0.15, 0.2, and 0.25 mg per drop tissue) were used and cultured in the same condition described in the methods. After being cultured for five days, the 3D-tissue models were fixed and immunostained by the CD31 antibody (specific marker for endothelial cells) whose fluorescence was monitored and observed by CLSM, as shown in Figure 2a. From the quantification data of the mean fluorescence intensity (Figure 2b), all of the conditions excepted the 0.15 mg of CMF showed a significantly lower CD31 staining, when compared to the condition without CMF. This phenomenon also happened for total branching points data (Figure 2c). Although the tissue model without CMF seemed to form similar blood capillary network than the 0.15 mg CMF one, the thickness of the tissue was later found much thinner as compared to the 0.15 mg CMF tissue model (Figure 2d), as measured on the histology data. These results showed that the CMF could act as a filler to provide a thicker tissue. Therefore, this higher thickness provided by the CMF induced significantly higher opened lumen structures of the blood capillaries, as shown in the tissue section of the histology data (red asterisk) in Figure 2d (2.31 ± 0.22 µm and 20.93 ± 0.55 µm of inner diameters for 0 and 0.15 mg CMF, respectively) [23].

### 3.3. The Role of NHDF for the Vasculature Formation

**Figure 3:**
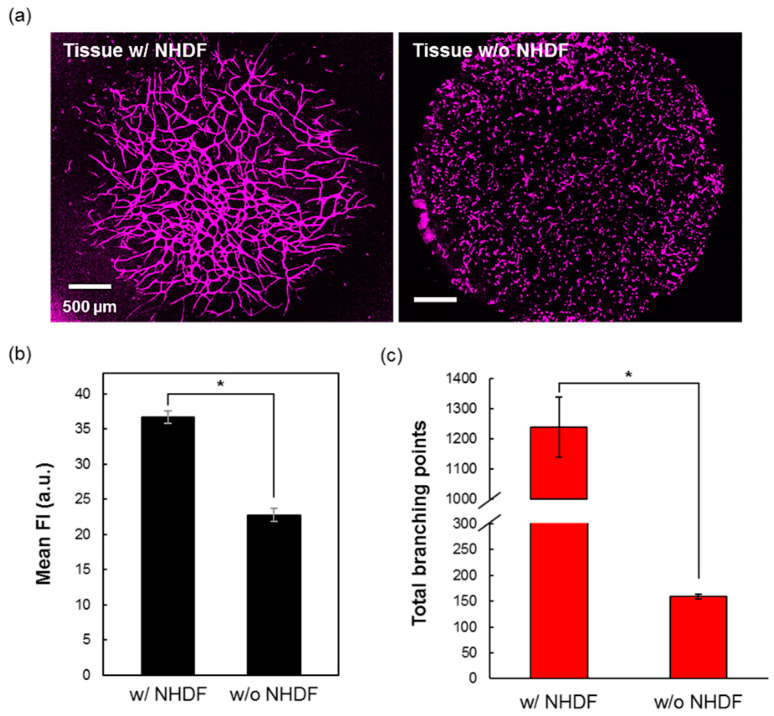
Normal human dermal fibroblast (NHDF) role for vasculature formation in CMF-fibrin gels. (**a**) Representative confocal images of 3D-blood capillary models with (left) and without NHDF (right). The blood capillaries were immunostained by CD31 antibody. Comparison between with and without NHDF in the 3D-blood capillary models by mean fluorescence intensity (**b**) and total branching points (**c**). Data are presented as mean ± s.d. (*) indicates that data are significantly different (*p* < 0.01).

NHDF is the fibroblast cells that generally support the vascular formation. For the next optimization step, we also investigated the necessity of NHDF in coculture with HUVEC in our tissues. The tissue was observed by CLSM and the image of the blood capillaries network were acquired, as shown in Figure 3a. From the image, the importance of NHDF for the vascular formation was clearly highlighted, the tissues without NHDF presenting almost only individual endothelial cells not connected in a vasculature network. The analysis of the mean CD31 fluorescence intensity of the tissue with and without NHDF then confirmed that the tissues with NHDF had higher vascular structures than the tissues without (Figure 3b). This result was supported by the total branching points analysis that also significantly showed a higher branching or networking for the tissues with NHDF compared to tissue without (Figure 3c). From this experiment, we could confirm the already reported important role of the NHDF as supporting cells for vascular formation [29,30].

### 3.4. Importance of the Cell Culture Medium Volume during the Culture

**Figure 4:**
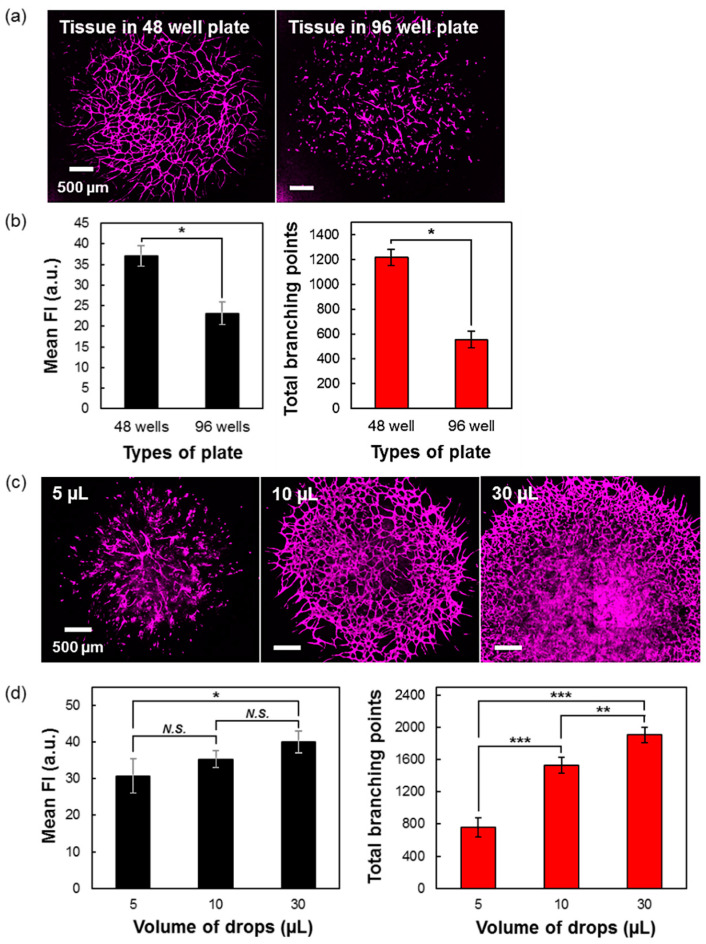
Comparison of cell culture medium and drops volumes for the optimized microarray assay culture of the 3D-blood capillary model. (**a**) Confocal CD31 images of 3D-blood capillary model in 48-well (left side) and 96-well (right side) plate and (**b**) their graph of mean fluorescence intensity and total branching points measured by ImageJ and Imaris software, respectively. (*) indicates that data are significantly different (*p* < 0.01). (**c**) Confocal images of the different volume of seeded drops (5, 10 and 10 µL) cultured in 48-well plate and (**d**) their quantification data of mean fluorescence intensity (MFI) and total branching points for each volume of drops. Data presented as mean ± s.d. (*, **, ***) indicate that data are significantly different (* *p* < 0.05, ** *p* < 0.01 and *** *p* < 0.001) calculated by ANOVA analysis. *N.S.* indicates no significant difference.

The ability to culture the 3D-blood capillary model was further investigated, in particular, for its use in microarray analysis. In this study, we used 48- and 96-wells plate to serve as medium and high-throughput assays, respectively. The 3D-tissues that were cultured in 48- and 96-wells plate were of the same volume (10 µL drop), but the available well volumes allowed to add more culture medium in the 48-well plate. After five days of culture, the tissues were observed by CLSM, as shown in Figure 4a, leading to 96-wells plate tissues showing a lower vascular formation network as compared to the tissues in the 48-wells plate. This might be due to the insufficient culture medium volume in the 96-wells plate (300 µL of culture medium), compared to the 48-wells plate (1.2 mL of culture medium), and this even when the culture medium in the 96 wells was changed every day. The quantification data confirmed that the mean fluorescence intensity and total branching points of tissues in 48-wells plate were significantly higher than that the 96-wells plate tissues in (Figure 4b).

### 3.5. Comparison of Different Volumes of Seeded Drops of 3D-Blood Capillary Model

As in the previous experiments, the 10 µL volume of drops was arbitrary chosen for the investigations, we then decided to optimize the volume of the drops by lowering (5 µL) and increasing (30 µL) the final drop volume. In this study, the conditions were kept constant, except for cells number. After five days of tissues culture, the 5 µL conditions were found with partially formed capillaries network, mainly in the center of the drops, as compared to the 10 and the 30 µL tissues (Figure 4c). This was proven by the mean CD31 fluorescence intensity and the total branching points data where the 5 µL tissues were significantly lower than the tissues with a volume of 10 µL (except mean CD31 fluorescence intensity) and 30 µL (Figure 4d). Although 30 µL tissues could also successfully form networks, the final diameter of the tissues was much larger when compared to 10 µL tissues. These larger tissues led to uneasy image acquisitions by confocal microscopy when using the high magnification of objective lens (4×), requiring several images to reconstruct the full tissue image. This fact makes the 30 µL drops model inconvenient and particularly unsuitable for the quantification analysis of the high-throughput screening process.

### 3.6. Time-Related Formation of Vascular Networks

**Figure 5:**
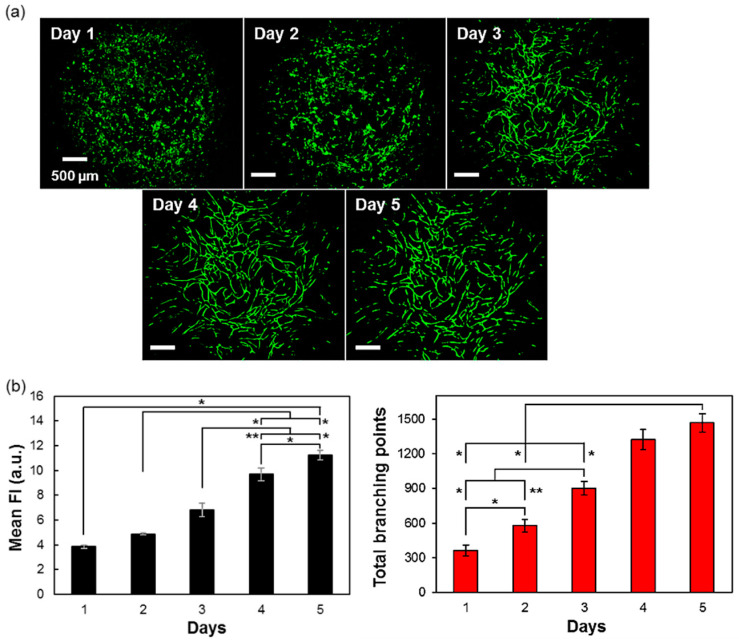
Time-related formation of vascular networks. (**a**) Confocal images of 3D-blood capillary models to show the formation of vascular networks during culture (Day 1–5), using green fluorescent protein labeled the EC (GFP-HUVEC) to live-image the formation of vascular networks without fixing the tissue. (**b**) Quantification data for mean fluorescence intensity (MFI) and total branching points during culture time. Data presented as mean ± s.d. (*n* = 2). (*, **) indicates that data are significantly different (* *p* < 0.05 and ** *p* < 0.01) calculated by ANOVA analysis.

Another step was to monitor the time effect on the vascular network formation during the five days of culture. In this experiment, we used green fluorescent protein labeled the EC (GFP-HUVEC) cells in order to enable visualization of daily formation of vascular networks in live without cell fixation. The confocal images that are shown in Figure 5a highlight the vascular network that started to form from day 3. This phenomenon was supported by quantitative data where the mean fluorescence intensity as wells as total branching points were increasing day by day (Figure 5b). As the time for culturing the tissues was relatively short, this will be an advantage point for preparing the 3D tissues that could be used as microarray analysis and, thus, be a powerful tool for screening assay.

### 3.7. Application of the Model in a Microarray Analysis

**Figure 6:**
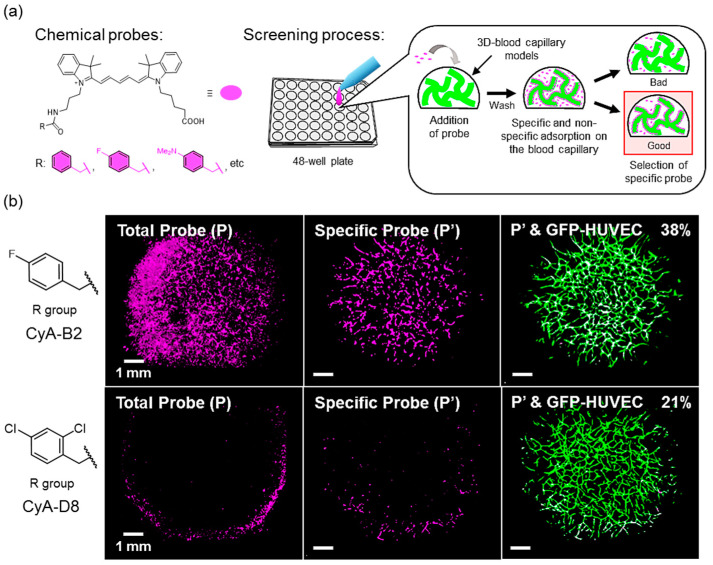
Application assay model (**a**) Schematic illustration of screening assay of chemical probes, differing in their R group (purple), using the 3D-blood capillary models prepared on the 48-well plate. (**b**) Confocal images of 3D-blood capillary models stained by two different probes, the top one being specific to the vasculature and the bottom one being unspecific.

Finally, the 3D-blood capillary models were used in an example of a microarray analysis. In our study, we used the 3D-blood capillary models to screen chemical probes until selecting the probe with the highest specific binding to the blood capillaries. The chemical probes were incubated on the 3D-blood capillary models and their live imaging was observed using CLSM, as illustrated in the Figure 6a [27]. The Figure 6b shows the examples of the live imaging of the tissues after staining with two different probes (CyA-B2 and CyA-D8). The confocal images (left image) show the total probe binding on the tissues (denoted as P), while the specific probe to the blood capillaries (denoted as P’, center image) which were obtained by Imaris software, using the area of the GFP signal from the HUVEC cells. The right image indicates the merged image between blood capillaries (GFP signal) and the specific probes (P’) in white. From this comparison, the probe CyA-B2 appeared with a higher specificity (38%) than the CyA-D8 (21%) and it can be used specifically for the specific live image of the blood vessels. This application of the microarray model confirmed in feasibility to be used in screening assays and it could be further applied for other types of tissues to provide reliable results when compared to 2D assay, especially for drug discovery application.

## 4. Conclusions

The micro-droplet of 3D-blood capillary models using collagen microfiber based fibrin gels were successfully fabricated and cultured in 48-wells plate to serve as a medium or relatively high-throughput assay. The blood capillary tissues were validated, and their reproducibility was proven by the quantification of their mean CD31 fluorescence intensity and their total branching points measured based on their confocal images. This method can be a powerful microarray assay for screening application, providing useful data due to their more physiologically relevance as compared to 2D assay. In addition to the imaging probes screening example that is shown here, another possible application of this model could be for drug discovery and screening of anti-angiogenic agents, blood vasculature being critical for tumor and metastasis progression in cancer patients.

## Supplementary Materials

The following are available online at https://www.mdpi.com/2072-666X/11/8/727/s1, Figure S1: Schematic illustration for preparation of 3D-blood capillary models using sedimentary culture and fibrin gels approaches, Figure S2: The sequence of the drop between solution 1 (thrombin with cells) and solution 2 (fibrinogen with CMF-50).
