# Supplementary material for: Fabrication of Blood Capillary Models for Live Imaging Microarray Analysis
Source: Micromachines (Basel). 2020 Jul 27;11(8):727. doi: 10.3390/mi11080727 (PMC7464508; doi:10.3390/mi11080727)
Supplement: Supplementary file 1 [file micromachines-11-00727-s001.pdf]

Fabrication of Blood Capillary Models for Live Imaging Microarray Analysis

Muhammad Asri Abdul Sisak, Fiona Louis, Sun Hyeok Lee, Young-Tae Chang and Michiya Matsusaki

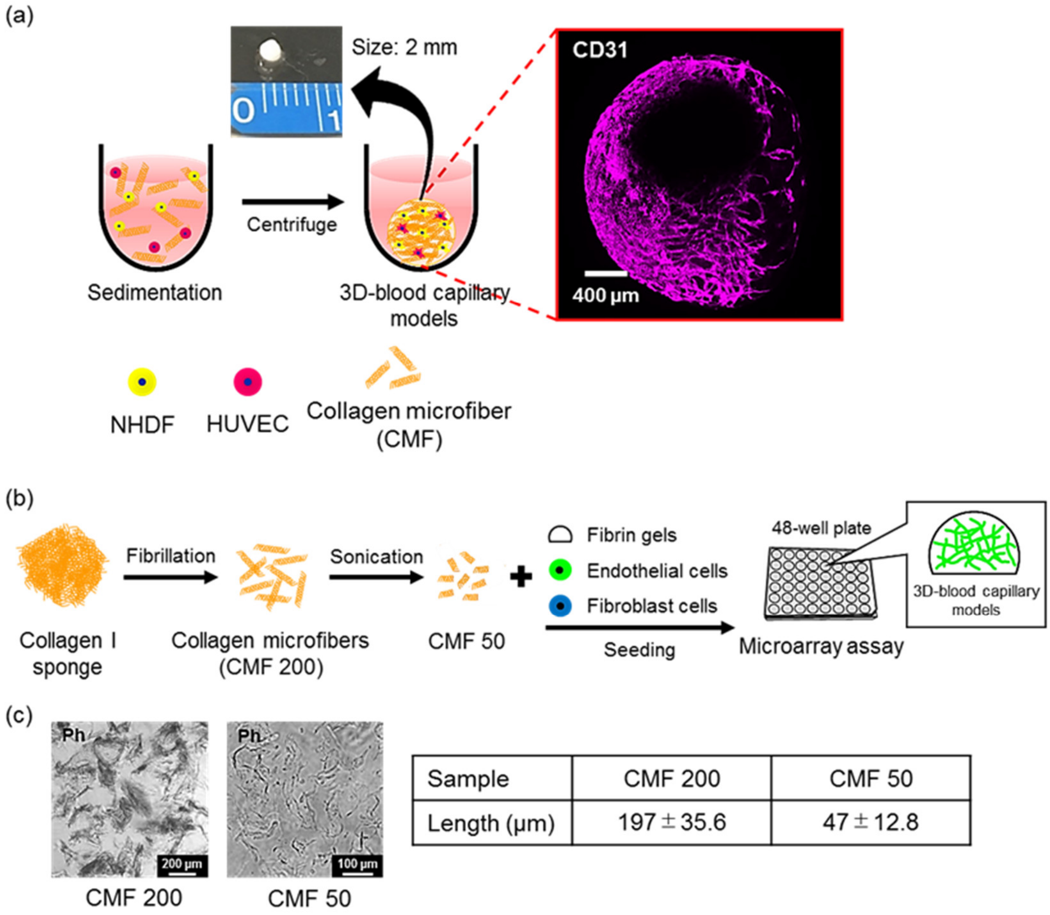

Sample	CMF 200	CMF 50
Length (µm)	197 ± 35.6	47 ± 12.8

Figure S1. Schematic illustration for preparation of three-dimensional (3D)-blood capillary models using 'sedimentary culture' and fibrin gels approaches (**a**) Preparation of 3D-blood capillary models using 'sedimentary culture' methods. The confocal image showed the dark area in the center of tissue balls. (**b**) Preparation of 3D-blood capillary tissues-based fibrin gels inside a 48-well plate for microarray assay. (**c**) Phase contrast images of CMF 200 and CMF 50 as well as their average length shown in the table. Data are presented as mean ± s.d. of 20 fibers were counted.

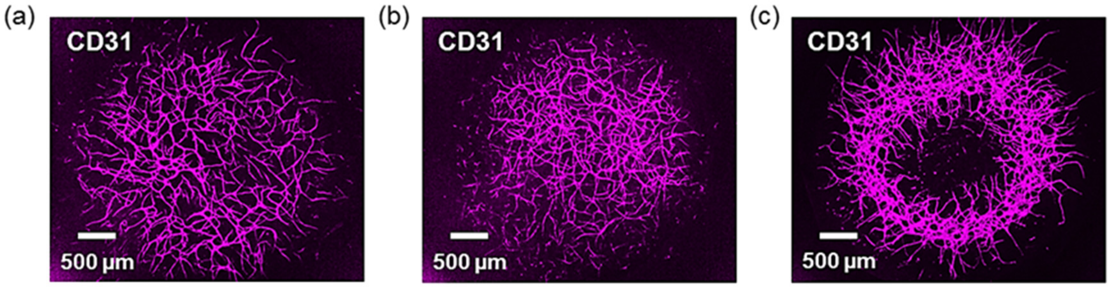

Figure S2. The sequence of the drop between solution 1 (thrombin with cells) and solution 2 (fibrinogen with CMF 50). (**a**) Solution 1 → Solution 2, (**b**) Solution 2 → Solution 1, and (**c**) Solution 1 → Solution 2 with mixing.
